# Dimensionality and dynamics for next-generation artificial neural networks

**DOI:** 10.1016/j.patter.2025.101231

**Published:** 2025-04-22

**Authors:** Ge Wang, Feng-Lei Fan

**Affiliations:** 1 Department of Biomedical Engineering, Department of Electrical, Computer, and Systems Engineering, Department of Computer Science, Center for Computational Innovations, Biomedical Imaging Center, Center for Biotechnology and Interdisciplinary Studies, Rensselaer Polytechnic Institute, Troy, NY, USA; 2 Department of Data Science, City University of Hong Kong, Kowloon, Hong Kong

**Keywords:** Artificial intelligence, AI, artificial neural network, deep learning, Transformer, dimensionality expansion, feedback loop

## Abstract

The recent awarding of the Nobel Prize in Physics to Geoffrey E. Hinton and John J. Hopfield highlights their profound impact on artificial neural networks. In this perspective, we explore how their foundational insights can drive the advancement of next-generation artificial intelligence (AI) models. We propose expanding beyond conventional architectures by introducing dimensionality through intra-layer links and dynamics via feedback loops. Network height and additional dimensions, alongside traditional width and depth, enhance learning capabilities, while entangled loops across scales induce emergent behaviors akin to phase transitions in physics. We discuss how these principles extend beyond transformers, fostering a new paradigm of intelligence inspired by physics-driven models and biological cognition mechanisms.

## The bigger picture

With Geoffrey E. Hinton and John J. Hopfield having been awarded the Nobel Prize in Physics, it is a good time to reflect on how to sustain the ongoing momentum in artificial neural network research. Achieving artificial general intelligence remains the holy grail, presenting numerous challenges that require novel insights to overcome. We underline that Hinton and Hopfield’s work has not only made historical contributions but will continue to have a profound impact on the development of artificial neural networks. From our perspective, their groundbreaking research suggests that new foundational architectures can be strengthened by incorporating links and loops within networks, aligning with the growing interest in surpassing Transformer architectures to enable next-generation foundational models that offer unprecedented feature representations and sophisticated emergent behaviors.

## Teaser

Building on the foundational work of Hinton, Hopfield, and others, this paper envisions the future of AI by integrating dimensionality and dynamics to enhance neural network performance and enable artificial general intelligence and even superintelligent behaviors. By incorporating multi-dimensional links and topologically sophisticated loops, future neural networks can achieve capabilities beyond the limits of current AI architectures. The insights presented here chart a pathway toward the next generation of AI.

## Main text

Since 2006, deep learning—pioneered by Hinton and others through innovations like the restricted Boltzmann machine—has flourished.^1^ We propose further generalization by introducing network height and even higher, more abstract dimensions through various links, such as Kolmogorov-Arnold Network (KAN)-type lines.^2^^,^^3^ Hopfield’s 1982 network model for human associative memory,^4^ which employs loops for dynamic evolution toward fixed points, serves as another foundation. More recently, physics-inspired models, including state-space models like Mamba,^5^ have leveraged noising-denoising, forward-backward, and compression-recovery loops to create foundational models for Bayesian inference, particularly via conditioned sampling for downstream tasks. These novel constructions of links and loops across space and time are poised to drive the next generation of artificial neural networks, shaping the future of artificial intelligence (AI).

The seminal work on the Transformer^6^ has come to dominate in research and applications of deep learning with phenomenal successes of foundation models^7^^,^^8^ such as ChatGPT, GPT-4o, and o1.^9^ Recently, it has been widely discussed how to surpass the current Transformer-based architecture toward next-generation AI models, especially foundation models. Although the Transformer, as the standard framework, cannot currently be replaced by non-Transformer architectures, the limits of Transformers are already well known. Transformers’ complexity does not fully support scaling law. In the foreseeable future, the most advanced GPUs will be based on 1 nm fabrication, approaching the atomic scale and meeting a physical ceiling to accommodate much larger models. Also, Transformers strongly rely on a large amounts of data, making them less effective in many tasks without troublesome fine-tuning. Therefore, surpassing the Transformer architecture represents one of the most promising directions. The recent works of this type include Mamba,^4^ KAN-type lines,^2^^,^^3^ the receptance weighted key value,^10^ and Mega,^11^ just to name a few.

**Figure 1:**
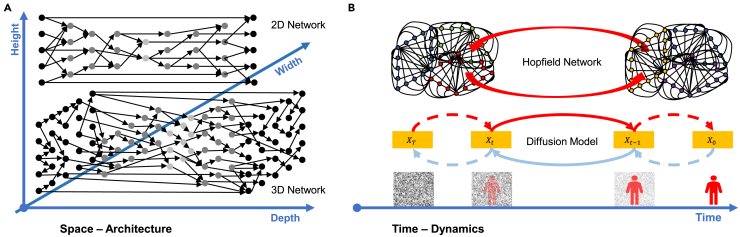
Future of artificial neural networks A 3D neural network with (A) width, depth, height, and potentially more dimensions to enhance deep learning spatially and (B) dynamics through feedback mechanisms to enhance deep learning temporally. A network empowered with links in higher dimensions and loops of more complicated topology would facilitate more advanced intelligent behaviors, such as phase transitions.

The Nobel Prize in Physics was recently awarded to Geoffrey E. Hinton and John J. Hopfield “for foundational discoveries and inventions that enable machine learning with artificial neural networks.” We underline that their work is not just important in the history of machine learning but will also have a far-reaching impact in the future, particularly relevant to the development of novel foundational architectures. By drawing insights from their works credited by the 2024 Nobel Prize in Physics, we believe that the novel use of links and loops in space and time would be the key to the future of the AI field at large, as illustrated in Figure 1^5^ to provide our conjectures into the future of deep learning and catalyze next-generation neural networks. Specifically, links represent dimensionality enhancement to enrich feature representations, and loops induce sophisticated dynamics, such as phase transitions, that enable the emergence of new AI model capabilities.

We emphasize that our perspective, which focuses on dimensionality and dynamics,^5^ differs significantly from other well-known perspectives, such as the fusion of rule-based and data-driven methods,^12^ embodied AI,^13^ quantum deep learning,^14^ and autonomous machine intelligence.^15^ Rule-based systems rely on explicit rules, while data-driven approaches leverage data to derive patterns and trends. Their fusion promises a balance between human expertise and machine intelligence. Embodied AI emphasizes interactions with the physical world to perceive the environment and take action. Quantum deep learning may revolutionize the computing paradigm, but its prime time is yet to come despite exciting technical developments over the past few years. In autonomous machine intelligence, Yann LeCun champions the concept of a world model, arguing that existing large models should enhance their capabilities in the perception-planning-action cycle. In contrast to these excellent and other perspectives on the future of deep learning, our perspective is unique and at the fundamental level of links and loops to form motifs, constructs, and architectures for superior intelligent performance.

## Introducing height and more dimensions

The formulation of backpropagation by Shun’ichi Amari^16^ paved the way for training multilayer neural networks. However, Geoffrey Hinton’s and others’ contributions to deep learning are also foundational and transformative, profoundly shaping the trajectory of AI research and its applications. These pioneers’ dedication to deep learning enabled learning intricate representations at scale and with flexibility, generating huge impacts on real-world applications. Their revolutionary insights initiated a dimensional outreach into the depth direction. In retrospect, further enriching feature representations through higher-dimensional augmentations promises to yield even greater advancements.

Indeed, naively deepening models may not be beneficial.^17^ In terms of expressivity, the width and depth of an artificial neural network are basically equivalent in principle, meaning that a wide network can be functionally transformed into a deep network, and vice versa.^18^ Practically, deepening a network often yields better performance than widening it, but a decent performance of a deep network requires a specific requirement of layer width. It has been proven that to approximate a function from *R*^*m*^ to *R*^*n*^, the minimum width required is *m* + *n*.^19^^,^^20^ The interplay between width and depth is intricate; for example, in Transformers, widening becomes necessary when deepening. If the width and depth are not balanced well, then increasing the depth would be inefficient or defective.^17^ It was demonstrated in Goyal et al.^21^ that a wider network with only 12 layers achieves performance comparable to that of a deep network with 30 layers. Throughput-wise, while a deeper network implies more sequential computations and higher latency, a wider network allows for easier parallelization.

The human brain operates as a three-dimensional (3D) network of adaptively interconnected neurons.^22^ This intricate network allows for complex processing of information and seamless integration of various cognitive functions. Given the complexity of human intelligence, it seems plausible that artificial networks should adopt a 3D structure to emulate the brain’s efficiency and versatility. First, the brain’s volumetric nature enables it to process and store vast amounts of information in a highly efficient manner. Neurons are densely packed in three dimensions, forming synaptic connections that facilitate rapid communication and parallel processing. Second, the brain’s organization is resilient and fault tolerant due to its redundant, entangled, and distributed nature, enabling it to withstand damage to individual components without catastrophic failure. Third, the three-dimensionality of the brain fosters the emergence of cognitive functions. The interplay between different regions of the brain, facilitated by its 3D connectivity, underpins our capacity for creativity, abstract thinking, and nuanced decision-making.

In addition to the width and depth of a deep artificial neural network, the third dimension of a network is height, which is largely overlooked in literature. By transitioning to 3D network models, we aspire to develop artificial systems that induce higher-order cognitive functions akin to those observed in the human brain. The height can be introduced through intra-layer links, as pointed out in recent studies.^23^^,^^24^ Shortcuts, which *bypass layers*, have worked well. Different from the commonly used shortcuts that connect layers, intra-layer links incorporate shortcuts *within a layer*, as shown in Figure 1A. The concept of height is a natural extension beyond the width and depth of neural networks. Simply wrapping a linear array of neurons into a 2D array does not change the fact that these neurons can be easily straightened out. On the other hand, intra-layer links implement neural interconnections within a layer so that neurons in the layer cannot be linearly unrolled, making the height direction non-trivial.

Introducing height is different from increasing depth in the following three senses. First, it increases neither the number of affine transformations nor the number of nonlinear activations, while increasing either width or depth does. Second, the mechanism behind intra-layer links involves breaking symmetry and reinforcing the mutual information among neurons within the same layer,^24^ thus reducing the hypothesis space of relevance. In Zhang and Zhou,^24^ intra-layer links are central (referred to as self-connections) in proving that spiking networks with these intra-layer links exhibit improved approximation capabilities and computational efficiency, i.e., modeling discrete dynamical systems with a polynomial number of parameters and a polynomial time complexity, although the concept of network height is not explicitly elaborated. Consequently, a narrower network with intra-layer links can possess the same capability as an exponentially wider network. For example, while the extended long short-term memory (xLSTM) replaced the scalar with a matrix to define cell states,^25^ the long short-term memory (LSTM) can be equipped with the height dimension to increase its memory capacity. Furthermore, like “depth separation,” we should consider “height separation.” This emphasizes the importance of height for network design, suggesting that a tall network can only be expressed by a short network with very large width and depth. It is also important to note that the width, depth, and height of a network could be flexibly converted for universal approximation, meaning that tall, wide, and deep networks can be transformed from one type to another. Coupling the width, depth, and height of a network empowers its performance without increasing the number of network parameters much. Therefore, instead of blindly increasing depth, we advocate for optimal balance among the width, depth, and height of a network to maximize its representation power and computational efficiency.

Since we exist in a 3D world, we can design neural networks beyond 2D configurations. We should seek a “dimensionality blessing” and avoid a “dimensionality curse.” For instance, in a 4D space, there are countless differential structures on 4-manifolds, whereas this is not the case in higher-dimensional spaces. Solving the Poincaré conjecture becomes notably simpler in spaces exceeding three dimensions, leading to a faster resolution of the high-dimensional Poincaré conjecture than the 3D counterpart. In mean-field theory, individual components, such as spins, of a system are assumed to interact not directly with their specific neighbors but with some averaged effect of the system. This approach replaces the complexity of local interactions with a mean field that encapsulates the behaviors of surrounding elements. As dimensionality increases, random fluctuations are mitigated through the averaging effect, which tends to cancel them out. As a result, the more interactions are aggregated, the smaller the impact of individual variations. Consequently, mean-field theory becomes more accurate when the dimensionality goes higher.^26^ Physicists are actively pursuing a theory that unifies standard models and general relativity, and it seems such a theory demands an 11D space. Unfortunately, in the machine learning field, the advantages of dimensionality often receive less attention than its drawbacks. In fact, increasing dimensionality has proven to be an effective strategy for improving feature separability, which is widely utilized in kernel methods. Anderson et al.^27^ demonstrate that a mixture of *m* Gaussians with equal known variance can be efficiently learned if *m* is lower bounded by the dimension *d*, implying that a sufficiently large dimension *d* is necessary to induce a favorable learning behavior. The crux of these findings lies in tensor decomposition. Distributing features across various axes can enhance the flexibility and decomposability of feature representation, thereby boosting learning effectiveness and efficiency.

The height dimension can be viewed or adapted as a dependency relationship between two networks: one is the main network and the other is the hyper-network interacting with the main network. In biology, the genotype encodes the phenotype, as seen from the fact that a human genome consisting of a small number of genes encodes the development of a human brain with a vast number of connections.^28^ Inspired by this idea, the hyper-network (genotypes) can be developed to design, construct, and optimize the main network (phenotypes). In other words, we can focus on relationships in the spirit of category theory.^29^ According to Yoneda’s Lemma^29^ in category theory, two categories, X and Y, are isomorphic if and only if their corresponding functors are isomorphic, where functors are structure-preserving maps between categories. Following this principle, we treat objects as indivisible atoms without an internal structure, meaning that each object is characterized by its relations to other objects. In this view, the entire system can be described by its associated functor.

Despite the merits of height or higher dimensions in general, incorporating them does not come at zero cost. The augmented hierarchy of higher dimensions may generally entail increased computational complexity and latency, leading to longer training and inference time. On the other hand, there may exist a potential "sweet spot" where the advantages of dimensional augmentation can render resultant networks more powerful and more efficient in certain tasks.

## Dynamics through feedback mechanisms

The Hopfield network is characterized by feedback loops, with the dynamics of interconnected neurons forming associative memory. As physics-inspired models, Hopfield networks employ an energy landscape to encode stored patterns at stable states and have the capacity to recall patterns even from partial information, like the human brain. This feedback dynamics can be likened to phase transitions, where the system evolves toward stable states, analogous to cognitive clarity in the brain. One of the most intriguing aspects of the brain’s function is its ability to undergo phase transitions, a phenomenon that has been observed in a wide range of physical systems, from water turning into ice to magnetization processes. Phase transitions occur through a sudden characteristic change of a sufficiently complex system with respect to a control variable, such as its temperature, pressure, or others. Such changes are often associated with the emergence of new collective behaviors that are not present in the system’s individual components. In the brain, phase transitions are thought to play a crucial role in the emergence of cognitive functions, such as learning, memory, and decision-making. Friston’s free energy principle in the cognitive science field^30^ assumes that any self-organizing agent must actively reduce disorder or uncertainty in the changing environment and constantly optimizes itself to include a built-in world model so that a “surprise” measure can be minimized that relates to the overall accuracy of the agent’s prediction or the expected outcome of the agent’s action. By this principle, intelligence emerges when the state of a self-organizing agent migrates from disorder to order, with its behavior seemingly purposeful and comprehensive.

We believe that an advanced intelligent neural network should also undergo phase transitions, which allow the mimicry of some essential brain functions. For example, just like the cognition status is transformed from confusion to clarity, a neural network should have a phase transition from a state of uncertainty to a state of confidence. Such phase transitions suggest that neural networks have learned in a way similar to how the brain works.

To harness the full potential of connectionism and engender phase transition behaviors, as shown in Figure 1B, we should still use feedforward links and, at the same time, embrace feedback loops. The Hopfield network and its variants^31^ rely on a feedback mechanism that fundamentally differs from a feedforward network and produces intriguing outcomes, such as associative memory. We should extend the Hopfield network to combine the feedforward and feedback mechanisms in innovative ways. Such a combination of links and loops would bring significant advantages. While the Hopfield network is featured by simple loops, the recently emerging diffusion-/flow-based and other physics-inspired generative models^32^ use multi-step loops. For example, the diffusion model allows for an original data distribution to be gradually noised into a featureless random field, and then incremental noise components can be step-by-step removed from the random field to sample the original data distribution (the feedback loop closed by the forward and reverse processes), which is also shown in Figure 1B. The whole noising-denoising loop can be done in either many (generic probability flow solutions) or single (consistency models) steps, leading to powerful dynamics with the solution existence, uniqueness, and stability established by the stochastic differential equation theory. An increasing number of studies report that the diffusion-/flow-based models set the state-of-the-art performance of generative AI, outperforming the famous generative adversarial network (GAN) and variational autoencoder (VAE) networks. In addition, while chain-of-thought^33^ focuses on linearity, tree-of-thought^34^ emphasizes branching and hierarchy, and graph-of-thought^35^ showcases connectivity and relationships, feedback loops enable the “loop-of-thought” process, a cyclical process where ideas are revisited and refined iteratively, allowing for ongoing introspection and retrospection, which can be seen as an advanced form of cognitive processing, allowing for a more dynamic exploration of ideas. As the latest example, the recently emerging structural state-space sequence models, such as Mamba,^4^ compress history to predict the future, and the relevant information is then selectively recovered in the future. The whole compression-recovering loop leads to dynamics for long-range dependence modeling.

From this point of view, a network that incorporates links in multiple dimensions and loops of complicated topology would behave more like our brain, giving rise to critical behaviors that have not yet been a focus of today’s AI research. Thus, future research should capitalize on sophisticated phase transitions, including Ising-type and topological phase transitions.^36^ Inspired by the Ising model, consisting of spin-up and spin-down polarities, in our recent study we flipped the gradient directions of neurons during network training to enhance robustness.^37^ Along this direction, topological phase transitions in physics should have counterparts in artificial neural networks. Topology describes properties of objects that remain unchanged under stretching or twisting but not tearing. The topological phase transition differs from a conventional phase transition. Therein, small vortices play a key role within a 2D or higher-dimensional space. At low temperatures, vortex/antivortex pairs form. As the temperature increases, such pairs disappear. Graphically, loops can be viewed as “holes” of a network, which can be regarded as a topological defect. The movement of vortices can be expressed as the transformation between links and loops. Through this transformation, a variety of topological phases can be manifested, and the rate of such transformation is analogous to temperature.

The proposed integration of intra-links and topological loops is fundamentally different from widely known baselines such as graph neural networks (GNNs^38^) or other advanced architectures. GNNs focus on learning structural relationships over graph data through message passing across edges. In contrast, our approach suggests the embedding of Hopfield-like dynamics directly into the architecture, introducing mechanisms for associative memory, energy minimization, state convergence, and phase transition within layers, sub-networks, or the entire network. Indeed, GNNs are primarily designed to propagate information between nodes along edges in a graph and generate node embeddings and edge predictions without inducing and utilizing neither internal dynamics nor memory. Again, our focus is on enhancing and empowering contemporary deep learning architectures with recurrent intra-layer/network interactions inspired by Hopfield’s work. These proposed mechanisms are not possessed by GNNs, which are designed for static graph-based learning tasks.

The enhancement of a neural network in both dimensionality and dynamics also facilitates AI for science. Demis Hassabis and John Jumper’s AlphaFold^39^ has exemplified the immense potential of AI for scientific discovery. However, a significant challenge remains; for example, how AI can deepen our understanding of highly complex nonlinear systems. In this context, reservoir computing models,^40^ such as echo-state networks^41^ and liquid-state networks,^42^ provide a promising framework for predicting nonlinear dynamic behaviors, including chaotic processes and extreme events. Reservoir computing takes advantage of the "blessing of dimensionality" by mapping input signals into high-dimensional feature spaces through a randomly initialized, fixed, highly nonlinear, and dynamic system. Then, a relatively simple yet trainable mechanism makes predictions based on the latent time-varying features extracted via these links and associated loops. We further posit that neural networks can serve as effective models for deciphering neural processes in the human brain, thereby enhancing our understanding of how neurons and neural circuits process information. In this context, the proposed 3D network, characterized by phase transitions, may become instrumental in elucidating biological intelligent behaviors, such as the critical dynamics of the brain, which is intrinsically 3D. This could also be crucial for the diagnosis, prognosis, and treatment of various neurological disorders.

Given the rapid momentum of AI model evolution, our above analysis suggests transcending the territory of the current network architectures from spatial and temporal angles along the directions revealed by Nobel Prize laureates Hopfield and Hinton, as well as other leading AI researchers. This perspective represents a convergence of classic and contemporary sciences with a central focus on the development of next-generation artificial neural networks. We welcome further brainstorming and collaboration.

## Declaration of interests

The authors declare no competing interests.
